# Can Family-Based Prevention be Successfully Implemented in Disadvantaged Primary Schools? Findings from a Pilot of Strengthening Family Connections in Australia

**DOI:** 10.1007/s10935-026-00933-7

**Published:** 2026-07-29

**Authors:** Meu Supol, Michele Burn, Louise McDonald, Keri Little, Michelle Benstead, Toni Gauntlett, Karina Tetkowski, Greta Small, John Toumbourou

**Affiliations:** 1 School of Psychology, Deakin University, Geelong, VIC 3220 Australia; 2 Meli, Geelong, VIC 3220 Australia; 3 ³Centre for Social and Early Emotional Development, School of Psychology, Deakin University, 221 Burwood Highway, Burwood, VIC 3125 Australia

**Keywords:** Family-based intervention, Family conflict, School attendance, Academic achievement, Prevention

## Abstract

This paper reports a pilot study evaluating the implementation of Strengthening Family Connections (SFC), the Australian adaptation of the Strengthening Families Program (SFP), a manualised family intervention recommended within international research. The study examined implementation along with changes in participant-reported outcomes over time, including child adjustment measured post-intervention and at 3- and 12-month follow-up. Families were recruited from 20 SFC groups delivered between 2012 and 2017 in disadvantaged primary schools in regional Victoria, Australia. A total of 125 parent–child dyads completed evaluation surveys at baseline and 52 remained in the study at 12-month follow-up. The children in the study demonstrated greater vulnerability than normative state samples of similar age. Those who remained in the study until the 12-month follow-up showed better baseline adjustment. Statistically significant changes were observed over time in parent-reported child school attendance (effect size [ES] = 0.40 standard deviation units), and child-reported antisocial peer affiliation (ES = 0.43) and family conflict (ES = 0.63). Based on non-random allocation, these changes were similar across the 8-session and 14-session versions, with some changes being greater with the 8-session version. Structural equation modelling revealed that reductions in family conflict from baseline to post-intervention were associated with lower antisocial peer affiliation at 3-month follow-up. Findings support the feasibility of implementing the Australian adaptation of the SFP in disadvantaged primary schools and document favourable changes in participant-reported outcomes over time. Controlled studies are needed to determine intervention’s effectiveness and to compare the relative effectiveness of the 8-session and 14-session versions.

## Introduction

High rates of child mental health problems have been observed internationally, with particularly severe problems amongst socioeconomically disadvantaged families (Lawrence et al., 2015). Family-based interventions have been proposed as a means of addressing risk factors in vulnerable families (e.g., Toumbourou et al., 2017). Family-based interventions are among the most effective treatments for child mental health disorders, including conduct disorders, anxiety disorders, and substance use disorders (Carr, 2018; Kumpfer & Alvarado, 2003; Hannan et al., 2000). Meta-analytic studies have found that many family-based intervention programs are more effective in reducing child behaviour and substance use problems than other forms of therapy (e.g., Baldwin et al., 2012; Dopp et al., 2017).

The *Strengthening Families Program* (*SFP*) (Kumpfer et al., 2016) is an internationally recognized evidence-based family-based intervention. The SFP was originally designed to selectively target high-risk families with drug-abusing parents and was found to be highly efficacious in randomized control trials, Cochrane reviews (e.g., Foxcroft et al., 2003; Spoth et al., 2007), and systematic reviews (Bulimwengu & Cartmel, 2022). It was also identified by the World Health Organization as one of the parent and child programs that could help reduce aggression in children (World Health Organization, 2010, p. 18). SFP initially targeted families with children aged 6 to 11 years (Kumpfer et al., 2016). Later applications were adapted for children of a wider age range (i.e., 0 to 17 years and 12 to 16 years) and were found to be effective in reducing risk factors and building protective factors to improve child mental health (e.g., increasing social competence; Kumpfer et al. 2002a).

*Strengthening Families 10–14* is a shorter (7-session) version that was developed and implemented in Iowa, United States (US). This version of the program is rated as well-supported by the Californian Evidence Based Clearinghouse for Child Welfare (2019) and as promising by Blueprints for Healthy Youth Development (2023) and Crime Solutions (2020). It has been reported to have high benefits relative to costs, with a cost return of $6 per $1 spent, as analysed by the Washington State Institute for Public Policy (2019), with specific benefits improving outcomes relating to crime, internalizing problems, externalizing problems, and problematic alcohol use. Cultural adaptations of the family-based intervention across families from varied backgrounds and ethnicities have also been found to be effective (e.g., African American, Asian, and American Indian populations; Kumpfer et al. 2002b; and Hong Kong population; Cheung & Chui, 2026). One of the current tasks of intervention science is to test programs evaluated as effective in the US in other international settings.

*Strengthening Family Connections* (*SFC*) is a version of the SFP that was specifically adapted for the Australian context (Burn et al. 2019a, b). The Australian adaptation was undertaken by the Barwon Child, Youth and Family service agency with the authorisation, consultation, and supervision of the SFP developer and owner, Professor Karol Kumpfer. The SFC program targeted families of children aged 8 to 12 years who were at high risk of mental health problems. Designed to target socioeconomically disadvantaged families, the SFC program was implemented in Australian regional primary schools that had high levels of socioeconomic disadvantage. This selectively targeted delivery of the SFC was expected to reduce child behaviour problems and peer conflict, thereby enhancing classroom learning opportunities (Domitrovich et al., 2010; Durlak et al., 2011). Overall, findings from the evaluation of the SFC pilot revealed a significant reduction in children’s emotional and behavioural difficulties and in parents’ psychological distress after program participation, with these changes sustained at 3-month follow-up (Burn et al. 2019a). Qualitative interviews with parents supported the program logic (Burn et al. 2019b).

### Impact on School Adjustment

 As the SFC was implemented in primary schools, it offered the opportunity to evaluate the association between program participation and child school adjustment. As prior evaluations of the SFP have focused on impacts on family adjustment and child behaviour, and only one has examined school bonding (Kumpfer et al. 2002a), the association with school adjustment remains unclear.

School adjustment is a multifaceted construct encompassing child participation in school-related activities, homework completion, academic achievement, and social interactions with peers (Jimerson et al., 2003). Improving school participation, academic achievement, and social relationships can have positive impacts on students, families, and communities. There are likely to be reciprocal relationships between family adjustment, child behaviour, and school adjustment. School adjustment problems (Kearney, 2008) are predicted by child mental health problems (Daniel et al., 2006), social disadvantage (Hancock et al., 2013), and family and peer problems (Domitrovich et al., 2010; Jaureguizar et al., 2013). School problems may exacerbate child behaviour and family difficulties (Kim et al., 1999) and predict further problems at school (Hancock et al., 2013).

These interactive influences of parents on child adjustment and school climate may be interpreted using the Social Development Model (SDM; Catalano & Hawkins, 1996, Chap. 4, p. 149; Haggerty & McCowan, 2018). The SDM is a specification of socioecological systems theory, which describes how adolescent behaviour is affected by social bonding within the interacting community environments of the family, peer group, and school. From this perspective, programs such as the SFC strengthen prosocial bonds by providing opportunities to build skills, which are strengthened through positive recognition. Across the course of adolescent development, strengthening prosocial family bonds are predicted to reduce adolescent antisocial behaviour and increase prosocial behaviour. When implemented within a school setting, these influences are expected to reduce peer problems, strengthen prosocial school involvement, and, in this way, enhance school climate.

### Family Conflict

A key focus of the SFP and SFC is to improve prosocial family bonds by reducing family conflict and enhancing family communication and positive interaction (Burn et al. 2019a). Family conflict is commonly measured through child reports of arguments, aggression, and conflict between family members (Arthur et al., 2002). Witnessing family conflict during childhood increases the risk of developing problems in adulthood, including mental illness (DeBoard-Lucas et al., 2010), substance abuse (Herrenkohl et al. 2012a, b), family violence (Costa et al., 2015) and educational failure (Supol et al., 2020). When compared to other family-based programs that target children aged 10–13 years, a systematic review conducted by Bulimwengu and Cartmel (2022) found that SFC demonstrated promising results in improving adolescent adjustment and parental distress.

Further research is warranted to establish how family conflict affects school adjustment (Supol et al., 2020). Family conflict may diminish parental support for homework and school tasks (Anderson et al., 2004; Stormshak et al., 2010). Alternatively, family conflict may act as a stressor, reducing school attendance (Chapman, 2007; McShane et al., 2001). Family conflict may also undermine school climate by exacerbating child behaviour problems, such as bullying of peers (Hemphill et al., 2012) and violence towards authority figures (Jaureguizar et al., 2013). Although longitudinal studies demonstrate that family conflict predicts school academic problems (Supol et al., 2020), the mechanisms as to how this occurs remain unclear. No prior evaluation has investigated whether reducing family conflict through programs, such as SFC, has flow-on effects on improving school adjustment.

Systematic reviews and meta-analyses support family-school programs as a means of improving student social behaviour, mental health, and academic achievement (Smith et al., 2021). The SFP has previously demonstrated reductions in family conflict and child behaviour problems (Brody et al. 2012; Burn et al. 2019a; Kumpfer and Alvarado 2003; Kumpfer et al. 2016), although no prior evaluations have investigated how these changes influence school adjustment.

*The current study* sought to address an important gap in the prior literature by assessing whether SFC program participation was associated with improved child school adjustment within disadvantaged primary schools. As previous evaluations of the SFP have been conducted with longer (i.e., 14-session; e.g., Kumpfer et al., 2003) and shorter (i.e., 7-sessions; Spoth et al., 2007) versions, the length of the SFC program was modified during routine service delivery and evaluated as a treatment factor in the present study. Prior SFC evaluations did not find outcome differences for longer and shorter versions at 3-month follow-up (Burn et al. 2019a).

It was hypothesized that at 12-month follow-up: (1) there would be improvements in child school adjustment over time, and (2) lower family conflict would predict improvements in child school adjustment.

## Method

### Study Design

An implementation evaluation design was selected in the context of translating an established program into a new country. We evaluated the routine implementation of the SFC pilot program using a repeated-measures pre-post design with 3-month (short-term) and 12-month (long-term) follow-up. Longitudinal surveys were completed both by parents and children. Participants were allocated to either an 8-session version or a 14-session version of the SFC program, based on their date of recruitment. The proportion of program sessions families attended was also examined to evaluate associations with program attendance. Structural equation modelling examined the effects of family conflict on child school adjustment. The implementation design focussed on evaluation within a routine service delivery setting and hence did not include design features such as randomised service allocation, which are important in efficacy evaluations. Data from longitudinal studies in state-representative populations were used to provide a normative non-intervention comparison group.

### Intervention: The Strengthening Family Connections program

The development and content of the Strengthening Family Connections program has been described previously (Burn et al. 2019a). In summary, the manualised program is skills-based and designed for up to eight families in each group, including both parents and children. Sessions were run weekly for 3 hours within the school setting during the school term, with curricula focussed on relationship, communication, conflict management, parenting skills, and alcohol and drug prevention. In the first hour, parent and child sessions ran concurrently in separate rooms, with two facilitators in each group. Parents and children then came together for a structured 45-minute parent–child session focused on parent–child interactions, positive play, and the practice of skills and family bonding. At the end of each parent–child session, families were provided with a meal to support the development of rituals and practices associated with preparing and sharing healthy food (Burn et al. 2019a).

The program was initially implemented as an 8-session version suitable for delivery within the Australian primary school term. An expanded 14-session version was subsequently developed and replaced the 8-session version, to increase mental health and family therapy content. The expanded parent sessions included psychoeducation on stress, depression, anxiety, and the influence of the family of origin. The child sessions included self-identity (i.e., dreams and wishes, gratitude, and strengths) and family history. The expanded parent and child sessions also included mindfulness exercises.

### Participants

Schools were targeted as a setting for family recruitment and program implementation based on disadvantage indicators, including being under-resourced and/or having significant welfare needs. A previous report provides details of the 72 families recruited from 2012 to 2015 (Burn et al. 2019a). In what follows, we report on a sample of 125 families who participated in the SFC program from 2012 to 2017 and who consented to complete the longitudinal evaluation surveys. The evaluation was designed to elicit responses from family dyads comprised of one parent reporting on one focal child within each family. Responses were not included from 12 parents in 12 families who provided a second survey nor for 20 children in 19 families who provided surveys for a second and, in one case, a third child.

### Procedure

The study was approved by the Deakin University Human Research Ethics Committee (DUHREC; no. 2012-231). After families had been accepted to participate in the program, parents were provided with a Plain Language Statement and signed a consent form to indicate informed consent for study measures. After consent from the school principal and teaching staff was obtained, families were recruited in 18 groups run in disadvantaged primary schools and 2 groups run in community centres in regional Victoria, Australia.

All children attending the school, aged 8 to 12 years, and their primary caregivers were invited to participate in the program regardless of welfare needs. After families had been accepted to participate in the program, they were provided with the Plain Language Statement. Where families provided informed consent, a battery of self-report questionnaires was administered to children and caregivers before the commencement of the intervention (T1), post-intervention (T2), and at follow-ups approximately 3 months (T3) and 12 months (T4) after program completion. Parents were given a $20.00 food voucher for completing the 3-month and 12-month follow-up surveys. Children completed surveys during the school period in a private room with a researcher present. Pre- and post-measures were completed in person and hardcopies of follow-up surveys were mailed out to parents with return postage-paid envelopes. The questionnaires took approximately 15 to 25 minutes to complete.

### Measures

#### Program and Demographic Measures

Participation in the 8-session (20%, 25 families) versus 14-session (80%, 100 families) of the SFC program was coded as a binary variable. The proportion of sessions attended by each family was also coded (*M* = 0.76, *SD* = 0.27). In terms of family demographics, the focus parent’s gender was coded, and 86% of the parents were mothers. Parents were aged 26 to 55 years (*M* = 39.2). Place of birth was coded, with all but 2.4% (3) born in Australia. Parents also reported on their education levels, with 68.8% reporting less than 12 years of secondary school education, and employment status, with 68.1% reporting no current employment. The focus child’s gender (58% boys) and age (*M* = 9.4 years, range = 7 to 12 years) were also recorded.

#### Parent Report Measures

*Schoolwork and school attendance* were measured using the Pediatric Quality of Life Scale (PedsQL; Desai et al., 2014). *Behavioural difficulties* were reported by parents using the Strengths and Difficulties Questionnaire – Parent Version (SDQ-P; Goodman, 2001). The SDQ is widely used in both clinical and community samples and has demonstrated sound psychometric properties (Warnick et al., 2008). Further details are provided in Table 1.

#### Child Report Measures

*Behavioural difficulties* were also self-reported using the Strength and Difficulties Questionnaire – Child Version (SDQ-C; Goodman, 2001). Several child-reported risk and protective factors, including individual-, peer-, and family-related factors, were measured using an adapted version of the Communities That Care Youth Survey (CTC; Arthur et al., 2002; Hemphill et al., 2011). These included *emotional control*,* coping*,* antisocial peers*,* family opportunities*,* family management*,* parent overcontrol*, and *family conflict.* A systematic review of 20 studies found the CTC survey to be a valid and reliable measure (Thurow et al., 2021). Further details are provided in Table 2.

**Table 1:** Parent-reported measures

Measure	What it measures	No. of items	Scoring	Example item	α
School work	Child difficulties with attention and keeping up with schoolwork (PedsQL)	2	5-point Likert scale (0 = *Never* to 4 = *Almost always*); reverse scored, with higher scores indicating greater difficulties	“My child is keeping up with schoolwork”	0.81–0.89
School attendance	Child school attendance problems due to health-related reasons (PedsQL)	2	5-point Likert scale (0 = *Never* to 4 = *Almost always*); higher scores indicate greater problems	“My child missed school because of not feeling well”	0.52–0.88
SDQ Conduct Problems; Hyperactivity; Emotional Symptoms; Peer Problems; Prosocial Behaviour; and Total	Child emotional and behavioural difficulties (SDQ-P)	25	3-point Likert scale (0 = *Not true* to 2 = *Certainly true*). Score ranges: Conduct Problems, Hyperactivity, Emotional Symptoms, Peer Problems, Prosocial Behaviour (5-item subscales, range 0 to 10). Total (sum of four sub-scales, range 0 to 40).	“Often loses temper”	0.82–0.86

Higher scores indicate greater difficulties unless otherwise specified. α values reflect internal consistency across study time points. PedsQL = Pediatric Quality of Life Inventory; SDQ-P = Strengths and Difficulties Questionnaire – Parent version; SDQ-P Total scores can be interpreted through the widely used bands: normal (0–13), borderline (14–16), and abnormal (17–40; Mellor, 2005)

**Table 2:** Child-reported measures

Measure	What it measures	No. of items	Scoring	Example item	α
SDQ Conduct Problems; Hyperactivity; Emotional Symptoms; Peer Problems; Prosocial Behaviour; and Total.	Emotional and behavioural difficulties (SDQ-C)	25	3-point Likert scale (0 = *Not true* to 2 = *Certainly true*). Score ranges: Conduct Problems, Hyperactivity, Emotional Symptoms, Peer Problems, Prosocial Behaviour (5-item subscales, range 0 to 10). Total (sum of four sub-scales, range 0 to 40).	“I am restless, I cannot stay still for long”	0.81–0.89
Emotional control	Ability to regulate emotions (CTC)	4	Yes (1) / No (0); higher scores indicate better emotional control #	“I know how to calm down when I am feeling nervous”	0.62–0.74
Coping	Cognitive and behavioural coping strategies (CTC)	4	Yes (1) / No (0); two items reverse scored; higher scores indicate better coping #	“I think about the best ways to handle it”	0.27–0.60
Antisocial peers	Association with peers engaging in antisocial behaviour (CTC)	5	5-point scale (0 = *None* to 4 = *Four of my friends*) ^	“Friends who have missed school without permission”	0.63–0.80
Bullying peers	Association with peers engaging in bullying students (CTC)	1	5-point scale (0 = *None* to 4 = *Four of my friends*) ^	“Friends bullied a student at school?”	-
Family opportunities	Perceived family support and involvement (CTC)	3	Yes (1) / No (0); higher scores indicate greater opportunities #	“My parents ask me what I think before family decisions”	0.33–0.64
Family management	Parental monitoring and rule clarity (CTC)	8	Yes (1) / No (0); higher scores indicate better family management #	“The rules in my family are clear”	0.41–0.73
Parental overcontrol	Perceived excessive parental control (CTC)	2	Yes (1) / No (0); higher scores indicate greater overcontrol #	“My parents try to control everything”	0.32–0.56
Family conflict	Frequency and intensity of family conflict (CTC)	3	Yes (1) / No (0); higher scores indicate greater conflict #	“People in my family have serious arguments”	0.41–0.73

α = Cronbach’s alpha, a measure of item-scale reliability at study time points. SDQ-C = Strengths and Difficulties Questionnaire – Child Version. CTC = Communities That Care Youth Survey. For each scale, higher scores indicate greater problems unless otherwise specified. # Scales represent item average, range 0 to 1. ^ Scale range 0 to 4

### Comparative Data

To provide normative data for the implementation trial, comparisons were made with two Australian state- or national-representative samples. The comparative samples had been longitudinally followed for 12 months or longer, providing trend data for children of similar age on the same measures. Variables from the comparative samples were coded to align with those in the SFC dataset, allowing meaningful comparisons of longitudinal trends.

Data were extracted from the International Youth Development Study (IYDS), which was designed to provide normative comparison data by recruiting state-representative samples of students in 2002 (McMorris et al., 2007). Cohorts were resurveyed annually in 2003 (Wave 2, Hemphill et al., 2011) and 2004 (Wave 3, Herrenkohl et al. 2012a, b). Waves 1 to 2 data from the IYDS, Victorian state, youngest (Grade 5) cohort were extracted for the current study comparisons. Due to non-measurement in Wave 1, comparative data for three measures were drawn from Waves 2 and 3.

Strengths and Difficulties Questionnaire (SDQ) Total scores were extracted from the Longitudinal Study of Australian Children (LSAC). The LSAC began in 2003 and recruited a birth cohort and kindergarten cohort representative of Australian children. Comparative SDQ data were extracted from both LSAC cohorts (Guy et al., 2016) to provide normative data for parent reports of Australian children at ages 8.5 years (T1) and 10.5 years (T2).

### Data Analysis

Data were analysed using STATA software. Temporal trends for the SFC sample were initially assessed by inspecting mean scores and standard errors at each survey time point (T1 to T4). Paired-sample *t*-tests were conducted to compare responses between T1 and T4, while regression analyses were used to predict responses at T4.

Comparative trends were examined by comparing the SFC children to baseline and longitudinal population norms. To enable these comparisons, means and standard errors were estimated, and effect sizes were calculated from baseline (T1) to longitudinal follow-up. Effect size differences and *p*-values were calculated to compare the 12-month trends for the SFC children with the longitudinal trends in the two comparator samples.

**Fig. 1:**
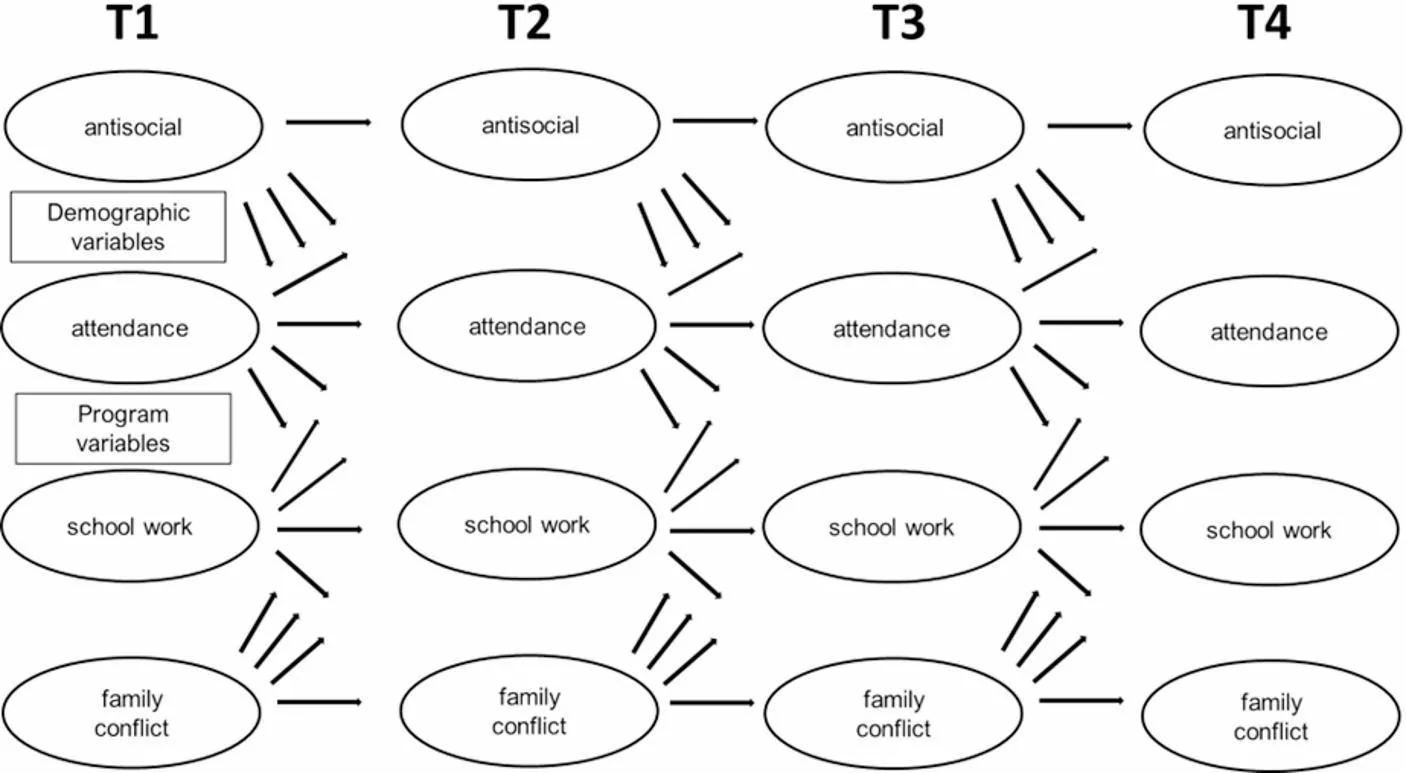
Theoretically predicted structural equation model. T1, baseline; T2, post-intervention; T3, 3-month follow-up; T4, 12-month follow-up; antisocial = antisocial peers; attendance = school health-related attendance problems

Structural Equation Modelling (SEM) was used to examine data from the SFC sample and assess the temporal relationship between child reports of family conflict and antisocial peers, as well as parent-reported school adjustment. The theoretically predicted structural equation model is presented in Fig. 1.

As outlined in Fig. 1, the predicted model anticipated that all constructs would be temporally stable and prospectively associated. Child reports of family conflict were anticipated to predict declines in parent-reported schoolwork and school attendance. Pearson correlations were examined and revealed no associations suggesting collinearity. The highest associations were between family conflict at post-test and 3-month follow-up (*r* = 0.61), schoolwork at pre- and post-intervention (*r* = 0.66) and at different time points for antisocial peers (*r* = 0.61 to 0.70) and school attendance (*r* = 0.67 to 0.73). Program variables (8- versus 1-session SFC version andthe proportion of sessions attended by each family) and demographic variables (parent gender, parent education, child age, child gender) were tested for fit, where they showed significant univariate correlations (*p* < 0.05) with the model constructs. Iterative model fitting continued under the guidance of modification indices until the observed data satisfied model fit criteria, with all predictors remaining significant. The model incorporating full missing data imputation (maximum likelihood with missing values) is reported; however, a model incorporating listwise deletion of missing cases was also completed as a sensitivity analysis.

Hypothesis testing was based on: (1) the *t*-tests evaluating whether significant improvements were evident in indicators of child school adjustment; (2) the SEM analyses evaluating whether reductions in family conflict predicted improvements in child school adjustment.

## Results

Results for mean changes over time are reported first, followed by the SEM results.

### Participants’ Changes Over Time: Temporal Trends

Table 3 presents data reported by parents and focal children across the four time points from baseline (T1) to 12-month follow-up (T4). The results were significant for fifteen of the *t*-tests (*p* < 0.05) comparing changes over time from baseline (T1) to 12-month follow-up (T4). With Bonferroni correction for 22 analyses (*p* < 0.0023), ten remained significant. The effect sizes ranged from small (0.06) to moderate (0.72). Regression analyses identified demographic and program variables associated with T4 outcomes; significant predictors are indicated in Table 3.

**Table 3:** Changes in parent and child reported variables from baseline (T1) to 12-month follow-up (T4)

Variables	T1		T2		T3		T4		T1–T4	T1vT4	SP
	Mean	SE	Mean	SE	Mean	SE	Mean	SE	ES	*p*	
**Parent Report**											
Schoolwork	1.75	0.07	1.52	0.12	1.44	0.14	1.15	0.17	-0.24	0.080	D. B, E, H
School attendance	0.90	0.08	0.71	0.11	0.61	0.10	0.46	0.12	-0.40	0.011	B, M, H
SDQ Total	16.08	0.72	12.77	0.60	11.12	0.83	9.29	0.84	-0.72	0.000	D. B, A
SDQ Conduct Problems	3.54	0.23	2.30	0.17	2.27	0.25	1.74	0.31	-0.62	0.001	D. B, A
SDQ Hyperactivity	5.54	0.25	4.89	0.23	4.34	0.36	3.56	0.33	-0.65	0.000	D. B
SDQ Emotional Symptoms	4.08	0.26	3.15	0.25	2.53	0.26	2.22	0.30	-0.52	0.002	B, A, F, E
SDQ Peer Problems	3.05	0.18	2.49	0.19	2.14	0.20	1.74	0.31	-0.38	0.015	D. B, E
SDQ Prosocial Behaviour	7.58	0.17	7.94	0.23	8.10	0.15	8.40	0.20	0.21	0.109	B
Sample Size	116		80		70		36				
**Child Report**											
Antisocial peers	0.57	0.08	0.49	0.10	0.34	0.07	0.31	0.08	-0.43	0.002	B, 8 S
Bullying peers	1.17	0.15	0.83	0.15	0.58	0.11	0.54	0.17	-0.48	0.003	8 S
Emotional control	0.61	0.03	0.73	0.04	0.76	0.04	0.77	0.04	0.35	0.007	B
SDQ Total	15.03	0.85	14.27	0.76	12.53	0.85	13.84	1.30	-0.07	0.321	B
SDQ Conduct Problems	3.44	0.26	3.01	0.22	2.42	0.25	1.94	0.28	-0.56	0.000	B, 8 S
SDQ Hyperactivity	4.64	0.30	4.48	0.24	4.04	0.35	3.96	0.41	-0.14	0.167	D. B
SDQ Emotional Symptoms	4.51	0.26	3.84	0.26	3.53	0.27	3.14	0.36	-0.52	0.002	B
SDQ Peer Problems	2.92	0.15	2.95	0.16	2.49	0.17	2.06	0.31	-0.42	0.002	D. B
SDQ Prosocial Behaviour	7.62	0.17	7.82	0.15	7.95	0.16	8.12	0.18	0.13	0.180	
Coping	0.48	0.05	0.60	0.05	0.60	0.06	0.65	0.08	0.31	0.016	8 S
Parent Overcontrol	0.55	0.04	0.50	0.04	0.42	0.04	0.38	0.05	-0.41	0.002	B
Family Opportunities	0.87	0.03	0.85	0.04	0.84	0.03	0.94	0.04	0.06	0.330	H
Family Management	0.86	0.01	0.88	0.02	0.92	0.01	0.91	0.03	0.20	0.083	
Family Conflict	0.53	0.05	0.44	0.05	0.34	0.04	0.31	0.05	-0.63	0.000	B, H
Sample Size	125		104		80		52				

T1 = baseline; T2 = post-intervention; T3 = 3-month follow-up; T4 = 12-month follow-up. T1–T4 ES = effect size measured from T1 to T4 in standard deviation units. T1vT4 *p* = *t*-test significance for T1 versus T4. SE = standard error. SP = significant predictors. D = significant *t*-test difference at baseline predicting T4 attrition. Significant regression predictors of variables at T4: B = T1 (baseline) score ; H = higher percentage of sessions attended; M = ,mother report; 8 S = 8-session version relative to 14-session version; E = lower parent education; A = older child age; F = female child)

#### Attrition Analyses

The *t*-tests revealed that adverse T1 (baseline) scores predicted higher T4 attrition for five parent-reported variables. For those missing at T4, scores were higher at T1 on schoolwork (T1: Retained Mean [M] = 1.44, SD 1.10 vs. Missing at T4 M = 1.89, SD = 1.71) and four SDQ externalising indicators – total (T1: M = 13.22, SD 7.40 vs. Missing M = 17.29, SD = 7.62), conduct problems (T1: M = 2.67, SD 2.11 vs. Missing M = 3.90, SD = 2.36), hyperactivity (T1: M = 4.89, SD 2.98 vs. Missing M = 5.84, SD = 2.73) and peer problems (T1: M = 2.51, SD 1.85 vs. Missing M = 3.29, SD = 2.18)) and two child-reported variables (SDQ hyperactivity (T1: M = 3.90, SD 2.89 vs. Missing M = 5.03, SD = 2.35) and peer problems (T1: M = 2.50, SD 1.77 vs. Missing M = 3.17, SD = 1.83)).

Regression analyses identified several factors associated with 12-month follow-up outcomes. Differences were observed between participants in the 8-session and 14-session versions of the program on four child-reported variables (antisocial peers, bullying peers, conduct problems, and coping), with more favourable outcomes reported in the 8-session group. Higher session attendance was associated with more favourable outcomes on parent-reported child schoolwork and school attendance, and child-reported family opportunities and family conflict. Lower parental education was associated with less favourable parent-reported child schoolwork, emotional problems, and peer problems at 12-month follow-up.

### Comparative Trends

Table 4 presents comparisons with available data from the two Australian normative samples of similar-aged children responding to the same measures. Table 4 revealed very small longitudinal changes in the comparison samples (effect sizes ≤0.05). Comparing the means in Table 4 with those in Table 3 showed that the SFC sample had higher problems and lower strengths at T1, based on non-overlapping standard errors. The exception was family conflict, which was higher in the IYDS sample at T1. The SFC sample improved from T1 to T4 more than the comparative samples at follow-up (FU) on all measures. Comparisons of the Table 4 effects with those in Table 3 (ESD) revealed significantly larger improvement effects for the SFC sample across all measures, except for coping, family opportunities, and family management. The SFC sample means changed from worse than the comparative samples at T1 to better than the comparative samples at FU on three measures: parent-reported SDQ Total, and child-reported emotional control and parent overcontrol.

**Table 4:** Comparative 12-month longitudinal trends for Australian children and effect size difference (ESD) comparing to Table 3 parameters

Variables	T1		FU		T1-FU	Difference	Difference
	Mean	SE	Mean	SE	ES	ESD	*p*
**Parent Report**							
SDQ Total	7.8	0.9(a)	9.7	0.9(a)	0.04	-0.75	0.000
Sample Size	~ 3400		~ 3300				
**Child Report**							
Antisocial peers	0.16	0.07	0.18	0.08	0.01	-0.44	0.002
Emotional control ^a^	0.97	0.10	0.65	0.10	-0.10	0.45	0.002
Coping ^a^	0.72	0.08	0.81	0.11	0.03	0.27	0.059
Parent Overcontrol ^a^	0.49	0.06	0.46	0.06	-0.02	-0.40	0.005
Family Opportunities	0.95	0.03	0.98	0.04	0.02	0.04	0.779
Family Management	0.89	0.02	0.92	0.02	0.05	0.15	0.293
Family Conflict	0.64	0.04	0.71	0.04	0.05	-0.68	0.000
Sample Size	914		890				

T1 = baseline. FU = follow-up (12-month follow-up for International Youth Development Study [IYDS, youngest cohort] and 2-year follow-up for Longitudinal Study of Australian Children [LSAC]). ^a^ Scores based on IYDS Wave 2 to 3, remaining scores Wave 1 to 2. SE = standard error (a) estimated. T1-FU ES = effect size measured from T1 to FU in standard deviation units. Difference ESD = effect size difference (Table 3 ES minus comparator Table 4 ES). Difference *p*-value for the ESD given the two sample sizes

### Structural Equation Models

**Fig. 2:**
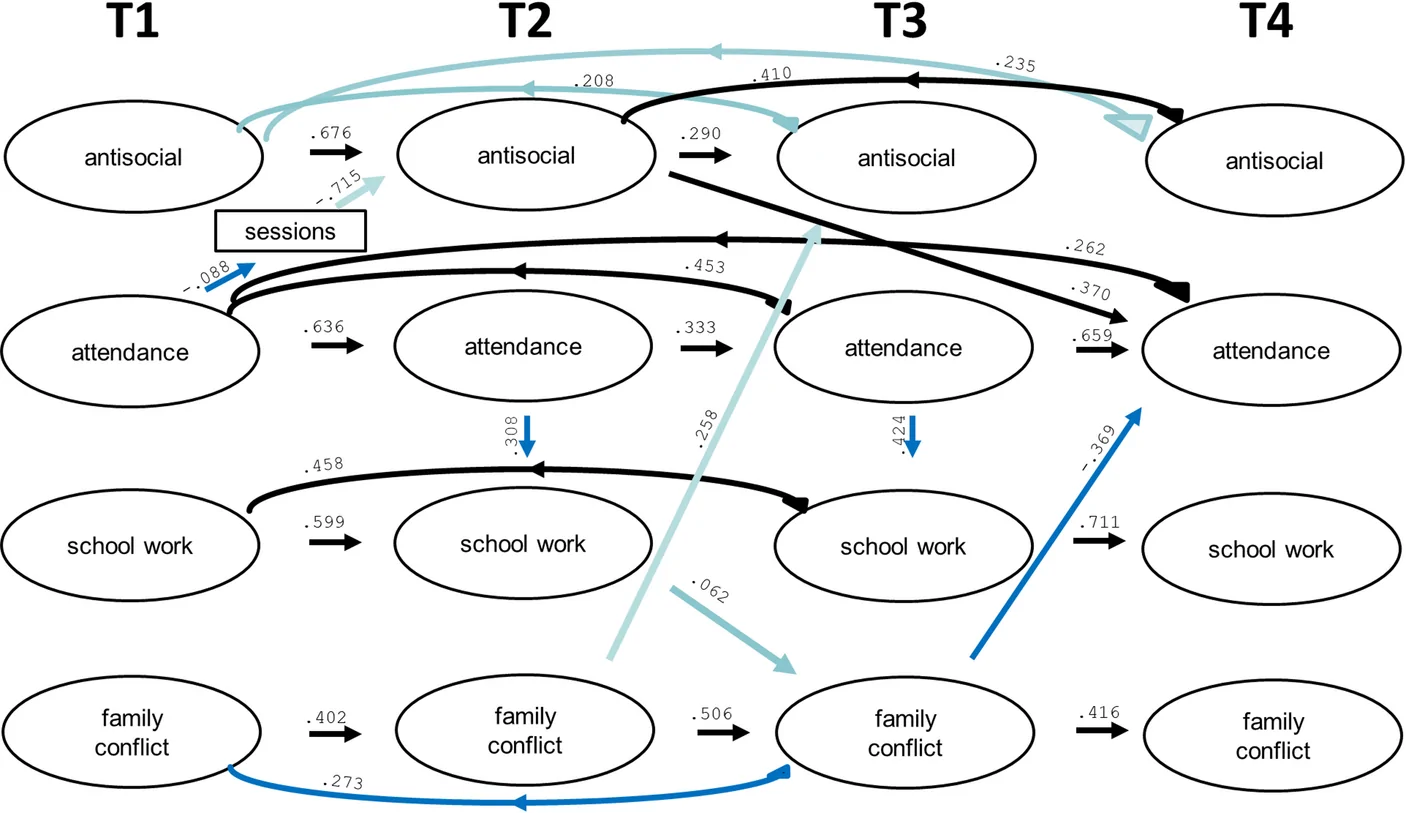
Observed structural equation model (*n* = 125). T1 = baseline; T2 = post-intervention; T3 = 3-month follow-up; T4 = 12-month follow-up. Arrows indicate significance: . antisocial = antisocial peers; attendance = school health-related attendance problems. Numbers are unstandardised coefficients. Due to the small sample longitudinal variables were not modelled as latent constructs but as average variable scores

The observed model is presented in Fig. 2.

In line with the predicted model (Fig. 1), the observed model (Fig. 2) showed considerable temporal stability across included constructs at different timepoints. There were few predictive associations between family conflict and schoolwork or school attendance. T3 child-reported family conflict predicted lower T4 parent-reported school attendance problems. Parent-reported school attendance did not predict schoolwork longitudinally but did show significant associations at T2 and T3. None of the demographic variables fit the model and the only program variable that fit was the proportion of SFC session attendance. Parent reports of higher T1 child school attendance problems predicted lower SFC session attendance, while higher SFC session attendance predicted lower child antisocial peer involvement at T2. T2 antisocial peer involvement predicted higher school attendance problems at T4 and T2 schoolwork problems predicted higher T3 family conflict. Family conflict at T2 predicted higher T3 antisocial peer involvement.

The final model (Fig. 2) was satisfactory based on fit criteria, *X*^2^ = 99.09, *p* = 0.59, RSMSEA = < 0.00, CFI = 1.00, AIC = 2331.438, BIC = 2520.935. As a sensitivity analysis, the same model was run with listwise deletion (*N* = 25), but not all predictors were significant. Three additional predictive relationships were required to satisfy fit criteria; T1 family conflict predicted lower T3 schoolwork and higher T4 family conflict, while session attendance predicted lower T4 family conflict.

## Discussion

This is the first study evaluating the implementation of Strengthening Family Connections (SFC), the Australian adaptation approved by the Strengthening Family Program (SFP) developer and owner, in Australia. In line with international trials, vulnerable families recruited from disadvantaged primary schools in this study reported favourable changes in child social and emotional adjustment over the 12-month follow-up period. These observed changes are consistent with patterns reported in previous evaluations of the SFP (e.g., Bulimwengu and Cartmel 2022; Kumpfer et al. 2002a).

With respect to the first hypothesis suggesting that there would be an improvement in child school adjustment indicators, Table 3 showed favourable changes in parent-reported schoolwork and school attendance and in child-reported school peer bullying between T1 to T4.

The second hypothesis suggesting that lower family conflict would predict improvements in child school adjustment was not supported. Child-reported family conflict has previously been conceptualised as a key proximal outcome in the successful implementation of the SFC program (Burn et al. 2019b). As anticipated, Table 3 indicated that child-reported family conflict was lower over time. However, the final SEM reported in Fig. 2 showed that family conflict did not predict schoolwork. Schoolwork problems post-intervention (T2) predicted family conflict at the 3-month follow-up (T3). Contrary to expectations, family conflict at the 3-month follow-up (T3) predicted lower school attendance problems at 12-months (T4). These findings are consistent with reciprocal associations between family conflict and school adjustment (Supol et al., 2020), and age moderation, suggesting the need to further examine modifiable moderating factors within these associations.

In addition to reduced family conflict, several additional patterns of change were observed among participating families. Table 3 showed that children whose families attended more SFC sessions reported greater increases in family opportunities. The SEM analysis (Fig. 2) further showed that greater SFC session attendance was associated with lower post-intervention (T2) antisocial peer involvement.

Several child outcomes were less favourable for families with lower levels of parental education attainment (Table 3). Parents with lower education levels reported worse outcomes in their child’s schoolwork, emotional problems and peer problems. Parents with lower educational attainment may reinforce a negative valuing of schoolwork and school attendance, or have fewer education resources to support school engagement and attendance, increasing opportunities for antisocial peer associations (Domitrovich et al., 2010). Parents’ low educational attainment may also exacerbate family stressors, increasing the risk of mental health problems (Lawrence et al., 2015). As most participating parents had low education, the school-based implementation model may provide an accessible mechanism for engaging families with lower educational attainment who are often underrepresented in preventive interventions (Burn et al. 2019b).

In line with the previous report of 3-month outcomes (Burn et al. 2019a), outcomes for the 8-session program were mostly similar to those observed for the 14-session program. Where significant differences were observed, child reports in the 8-session program were superior at 12-month follow-up (T4) for antisocial peers, bullying peers, conduct problems, and coping. However, because allocation was not randomized and the study lacked a control condition, these findings should not be interpreted as evidence regarding the necessity or therapeutic value of specific program components. Rather, they indicate that a shorter version can be delivered feasibly while observing outcome patterns comparable to those observed in the longer version.

The comparisons presented in Table 4 provided normative Australian data that can be compared with the recruited SFC sample (Table 3). These comparisons revealed that the SFC sample tended to be vulnerable at baseline, based on lower scores compared to state-representative normative samples of Australian children. These findings support the recruitment methods used in this implementation trial. Improvements from baseline to 12 months later tended to be significantly larger in the SFC sample than for children in the normative samples. This reduces concerns that the findings solely reflect repeated measurement effects.

The current findings demonstrate congruence with several aspects of the Social Development Model (SDM; Catalano & Hawkins, 1996, Chap. 4, p. 149; Haggerty & McCowan, 2018). The SEM findings were consistent with several predictors of the SDM by demonstrating mostly temporal stability in family, peer and school adjustment across adolescent timepoints. Consistent with the SDM, the children’s participation in SFC sessions, that focussed on prosocial relationship and communication skills was associated with reports of reduced family conflict (Table 3). Prior research suggests that exposure to family conflict increases the likelihood of the child’s antisocial or aggressive behaviour (Estévez et al. 2018; Hemphill et al. 2012; Herrenkohl et al. 2012a, b). Lower levels of post-intervention family conflict in the current study, which is consistent with prior SFP evaluations (see Foxcroft et al. 2003; Kumpfer et al. 2002a, b, 2016; Spoth et al. 2007), was associated with lower antisocial peer affiliations at 3-month follow-up. The observed associations are broadly consistent with propositions of the SDM concerning links between family relationships and peer affiliations.

The available evidence suggests the intention to recruit disadvantaged and vulnerable families was successful. The SFC selectively targeted disadvantaged schools, where all families were invited to participate. The analytic sample revealed 68.8% of parents reported less than 12 years of secondary school education and 68.1% were not involved in any form of employment. The average baseline (T1) score for parent- and child-reported total child difficulties (as measured on the SDQ; Goodman, 2001) was in the ‘borderline’ classification, suggesting the average child participant may have had clinically significant difficulties. This classification indicates the presense of some problematic symptoms, although a clinical diagnosis was not necessarily warranted (Domitrovich et al., 2010; Flay et al., 2005).

### Study Strengths and Limitations

A strength of this study was the novel investigation of feasibility in the Australian disadvantaged primary school context. The study evaluated implementation and outcomes within routine service delivery, used validated longitudinal surveys and included both child- and parent-reported assessments across a 12-month follow-up. Normative longitudinal data provided a novel comparison.

The current study has several limitations. The implementation evaluation did not include a no-treatment control group. The observed effect sizes are of a similar magnitude to those reported in previous controlled SFP trials where improvements were significantly greater relative to controls, and similar improvements were not observed in comparable Australian longitudinal studies with students of the same age followed for 12 months or longer (Table 4). However, given our implementation trial design, we cannot attribute the observed improvements to intervention effects and cannot make a strong causal inference.

Another limitation concerns attrition. There was substantial attrition at the 12-month follow-up, and attrition analyses indicated that participants retained in the study generally exhibited more favourable baseline functioning on several indicators than those lost to follow-up (Table 3). This pattern of selective attrition increases the possibility that observed changes at later assessments may overestimate the outcomes that would have been observed had all participants remained in the study. Consequently, findings from the 12-month follow-up should be interpreted cautiously and viewed as descriptive of participants who completed follow-up assessments rather than representative of all families initially enrolled in the program. Together with the lack of a control group, selective attrition limits the strength of conclusions that can be drawn regarding longer-term participant outcomes. Given that child externalizing problems was one of the key indicators for attrition, additional resource allocation and targeted assistance may be warranted to maintain contact with families experiencing these problems in routine service delivery.

As an evaluation in a routine service delivery context, recruitment and follow-up continued over several years. This increased opportunities to recruit participants and capture temporal change. The extended time period for service delivery may have cost implications that would benefit from economic evaluation. Within the routine service delivery evaluation, there was no exclusion of parents and children with an intellectual disability, severe mental illness or personality disorders which may have weakened the effect sizes by reducing the reliability of self-report data. However, by including a range of children, the sample is more representative of the typical recipients of such services.

A further limitation is that the comparison between the 8- and 14-session versions was observational rather than experimental. As participants were not randomly allocated and the two versions were delivered at different stages of implementation, the 14-session version being delivered after the 8-session version, the study was not designed to determine whether one version was more effective than the other. The findings are therefore best interpreted as describing different outcome patterns observed under changes in routine implementation conditions.

The child age range (7 to 12 years) in the current sample was at the lower end for reliable self-report. This may partly explain why several child-report variables had low internal-consistency (alpha) within the current sample at one or more follow-up timepoints including: coping, family opportunities, family management, parent overcontrol, and family conflict. Structural models confirmed the temporal reliability of these scales. Specifically, the SEM analyses presented in Fig. 2 demonstrated family conflict had significant temporal stability, with wave to next wave coefficients equating the other scales in the model. Additional SEM analyses were completed for all the above scales. These models had a good fit and demonstrated significant temporal stability from wave to next wave for all scales and proximal timepoints.

Finally, based on the aims of the current study, the structural equation model was fitted to schoolwork by adjusting for known relationships. Therefore, paths between family conflict and other outcomes were not systematically tested in the model, and some may have been overlooked (e.g., the path between family conflict and behaviour and emotional outcomes), where they were not recommended by modification indicators. The measure of school attendance was limited to measuring only a selection of child health-related school absences observable by parents.

### Implications and Future Directions

Findings from this pilot implementation study suggest that the 8-session SFC format can be delivered feasibly within disadvantaged Australian primary schools. The program recruitment strategy through disadvantaged primary schools appeared to successfully target vulnerable families (based on a comparison between Tables 3 and 4).

The current study demonstrated that it was feasible to recruit vulnerable children and documented favourable changes in emotional and social adjustment measures among participating children, signalling an important target for future family-school interventions. The present study is one of few to examine effects across parent and child-report measures that are relevant to child school adjustment. We observed small positive changes in parent reports of children’s schoolwork and school attendance. An important finding was that children reported reductions in involvement in peer antisocial behaviour and bullying from pre-to 12-month follow-up. If confirmed in controlled studies, these observed changes could have implications for broader school climate and peer relationships. School-wide effects have previously been demonstrated through interventions delivered to targeted families within the Australian secondary school setting (Toumbourou & Gregg, 2002). To study whether benefits extend across disadvantaged primary school populations, it is recommended that the SFC be more widely implemented within a larger cluster-randomized controlled dissemination study.

Family opportunities and family management did not significantly improve at T4 (Table 3), and longitudinal trend effects were not significantly different to comparative samples (Table 4). These findings suggest that the SFC content could be strengthened to improve these outcomes, which are theoretically important according to the SDM.

The finding that family conflict did not directly predict schoolwork suggests that there may be indirect mechanisms at work. This study found that family conflict increased antisocial peer involvement and paradoxically reduced school attendance problems at the 12-month follow-up. These findings have implications for theoretical frameworks and future research regarding the effects of family conflict on academic outcomes. Prior reviews have noted a range of mediating and modifying factors (Supol et al., 2020). The current findings suggest that effects may be reciprocal in some contexts and modified by children’s age and the frequency and intensity of family conflict. The current findings are consistent with SDM propositions that, when implemented in a school system, reductions in family conflict may be relevant to broader school outcomes such as peer bullying and antisocial behaviour and warrant further investigation in controlled studies.

## Conclusions

This study contributes evidence regarding the international implementation of the Strengthening Families Program, specifically the Australian-adapted Strengthening Family Connections version, in disadvantaged primary school settings. The findings indicate that recruitment and engagement of vulnerable families through disadvantaged primary schools was feasible and a range of favourable changes in parent- and child-reported outcomes were observed over the 12-month follow-up period. Higher session attendance was associated with greater changes in schoolwork and school attendance. However, the absence of a control group and substantial attrition, particularly at 12 months, mean that these findings should be interpreted cautiously and not as evidence of intervention effectiveness. Outcomes observed in the 8-session version were broadly comparable to those observed in the 14-session version, suggesting that the shorter version warrants further investigation in future controlled implementation and effectiveness studies.
